# Possible pathogenicity of commensal *Entamoeba hartmanni* revealed by molecular screening of healthy school children in Indonesia

**DOI:** 10.1186/s41182-018-0132-7

**Published:** 2019-01-15

**Authors:** Takahiro Matsumura, Joko Hendarto, Tetsushi Mizuno, Din Syafruddin, Hisao Yoshikawa, Makoto Matsubayashi, Taro Nishimura, Masaharu Tokoro

**Affiliations:** 1 Department of Parasitology, Graduate School of Medical Sciences, Kanazawa University, 13-1, Takara-Machi, Kanazawa, 920-8640 Japan; 2 Department of Medical Technology and Clinical Engineering, Hokuriku University, Kanazawa, 920-1180 Japan; 3 Department of Public Health and Preventive Medicine, Faculty of Medicine, Hasanuddin University, Makassar, 90245 Indonesia; 4 Malaria and Vector Resistance Laboratory, Eijkman Institute of Molecular Biology, Jakarta, 10430 Indonesia; 5 Department of Parasitology, Faculty of Medicine, Hasanuddin University, Makassar, 90245 Indonesia; 6 Department of Chemistry, Biology, and Environmental Sciences, Faculty of Science, Nara Women’s University, Nara, 630-8506 Japan; 7 Department of Veterinary Science, Graduate School of Life and Environmental Sciences, Osaka Prefecture University, Osaka, 598-8531 Japan; 8 Department of Clinical Trial and Clinical Epidemiology, University of Tsukuba, Tsukuba, 3058577 Japan

**Keywords:** *Giardia intestinalis*, *Entamoeba hartmanni*, Pathogenicity, Asymptomatic infection, Diarrhea

## Abstract

**Background:**

Although parasites are still endemic in developing areas, residents in those regions seem not to be affected by the presence of intestinal protozoans. This study aimed to investigate whether pathogenic and commensal protozoans are the causal agents of diarrhea via a school-based cross-sectional survey conducted in Indonesia, in September 2016.

**Results:**

Molecular screening for intestinal protozoans in collected 144 stool samples from healthy students (age range 7–15 years) was carried out. The prevalence of protozoan parasites was as follows: *Giardia intestinalis* (56.3%), *Entamoeba histolytica* (0%), *E. dispar* (6.9%), *E. moshkovskii* (0%), *E. hartmanni* (31.3%), and *E. coli* (44.4%). Observational evaluation of stool conditions using the Bristol stool chart confirmed the loose stool rate (33.3–90.9%) in each age group. Logistic regression analysis of protozoan infection or colonization for loose stool (mild to severe diarrhea) as an outcome revealed no significant findings in examined protozoans including pathogenic *G. intestinalis* infection [adjusted odds ratio (AOR) 0.78, 95% confidence interval (CI) 0.36–1.67], except in *E. hartmanni* colonization (AOR 2.81, 95% CI 1.1–3.7, *P* = 0.026).

**Conclusions:**

The molecular survey of intestinal protozoans targeting healthy population with their stool form evaluation could address the pathogenicity of those parasites appropriately. In comparatively higher-age children at least 7 years of age or greater in the endemic area, *G. intestinalis* could regard commensal, while *E. hartmanni* seems to possess a certain pathogenicity as a causal agent of mild diarrhea.

## Background

Since *Giardia intestinalis* (also known as *G. lamblia* or *G. duodenalis*) infection is still highly endemic in most developing regions [1], the burden of those infections are considered a public health issue, especially among children in rural communities. Indeed, various harmful outcomes related to *G. intestinalis* infection, including significantly lower weight for age z-score (WAZ) and weight for height z-score (WHZ), vitamin A deficiency [2], decreased serum iron and zinc content [3], dual burden of overweight/obesity and stunting [4], and higher burden of intestinal schistosomiasis and related anemia [5], have been epidemiologically estimated.

However, certain studies have reported contrasting findings. Goto et al. reported that pathogenic outcomes of *G. intestinalis* infection were only observed during initial exposure [6]. The correlation between WAZ and/or WHZ and *Giardia* infections was confirmed using anti-*Giardia* IgM; however, this correlation was no longer observed upon an increase in IgG titers. Moreover, a significant correlation between higher socioeconomic status and low Giardia infections [7] suggests the presence of socioeconomic bias on the above-mentioned harmful outcomes, since these outcomes are also considered to be the results from poverty.

To address the burden of *G. intestinalis* infection on children living in highly endemic regions, it is probably important to determine whether the infection causes diarrhea, especially beyond outside of medical facilities even in the endemic areas, though limited information is available regarding this aspect.

In contrast to *G. intestinalis*, various commensal intestinal protozoans have been comparatively neglected; however, recent molecular epidemiological studies have addressed newly defined potentially pathogenic intestinal protozoan parasites such as *Entamoeba moshkovskii* [8] and *Dientamoeba fragilis* [9], both which have been considered non-pathogenic for many years. The presence of morphologically indistinguishable pathogenic *E. histolytica* and non-pathogenic *E. dispar* seemingly prevented the determination of the pathogenicity of *E. moshkovskii* for a long time, while the difficulty of detection, owing to the comparatively small and fragile trophozoites, which are usually only excreted in stool, might serve as an obstacle to detecting *D. fragilis* itself in patient stool samples. This is also the case for other *Entamoeba* species, such as *E. coli* and *E. hartmanni*, which are generally highly endemic in tropical and sub-tropical rural areas [10]. Their examinations have rarely been conducted primarily, since they have been considered non-pathogenic.

In this study, we evaluated the pathogenicity of *G. intestinalis* and commensal *Entamoeba* species. To avoid socioeconomic and sampling biases, healthy school children in a small village were recruited, and correlations between diarrheal stool form and parasitic infections or colonizations were assessed.

## Materials

### Study design

To analyze the pathogenicity of *G. intestinalis* and non-pathogenic *Entamoeba* spp., a cross-sectional, school-based sample collection was conducted in September 2016 at the Kera-Panba elementary and junior-high school (location: 9° 38′ 36.13″ S, 119° 0′ 58.05″ E), Wainyapu village, Sumba Islands, Indonesia.

### Sample collection and evaluation of stool form

We collected 144 stool samples from healthy school children (74 males; age range, 7–15 years; median age, 10.0 years), using stool bags. Stool form was evaluated using the Bristol stool chart [11], based on a seven-point scale as follows: 1, separate hard nut-like lumps; 2, sausage-shaped but lumpy; 3, sausage- or snake-like but with cracks on the surface; 4, sausage- or snake-like, smooth, and soft; 5, soft blobs with distinct edges; 6, fluffy pieces with ragged edges, a mushy stool; 7, watery, no solid pieces. In this study, the criteria for stool form were determined in accordance with the aforementioned scale as follows: constipation (score, 1–2), normal stool (score, 3–4), and loose stool (score, 5–7).

### Polymerase chain reaction (PCR)-based DNA sequencing survey of intestinal protozoans

From the stool bag, 0.2 g of stool samples were preserved with 600 μL of DNAzol® reagent (Molecular Research Center, Inc., Cincinnati, OH, USA) at 4 °C for further analyses. Genomic DNA was extracted from DNAzol®-treated sample mixtures in accordance with the manufacturer’s instructions with slight modification, as described previously [12]. The final products were suspended in 80 μL of 10 mM Tris-HCl (pH 8.0) containing 1 mM EDTA and preserved at − 30 °C for subsequent analyses.

Extracted DNA was screened via PCR targeting the 18S small subunit of ribosomal RNA gene (18S rRNA) locus of *G. intestinalis* and subsequent genotyping for assemblage (ass.) A and ass. B [1]. The 18S rRNA locus of *Entamoeba* spp. (*E. histolytica*, *E. dispar*, *E. moshkovskii*, *E. hartmanni*, and *E. coli*) were also screened via a universal nested PCR and subsequent species-specific PCRs [10].

### Statistical analysis

We used multivariate logistic regression modeling to examine the association between the loose stool form (binary value) as an outcome and the explanatory factors such as age (continuous value of years), male (binary value vs. female), and the molecular screening results of positives for those target protozoans and genotypes (binary value). The association between the loose stool form and the summarized binary values such as *G. intestinalis* infection and any amoeba infection (at least one amoeba detected samples) was independently analyzed with other demographic data (age, male). Statistical analyses were conducted using R version 3.3.3 (2017-03-06) [13] with R package “epiDisplay” (version 3.2.2.0) [14]. Differences in variables were analyzed using the Wald’s test, with *P* < 0.05 indicating statistical significance.

## Results

### Results of stool form evaluation

**Fig. 1:**
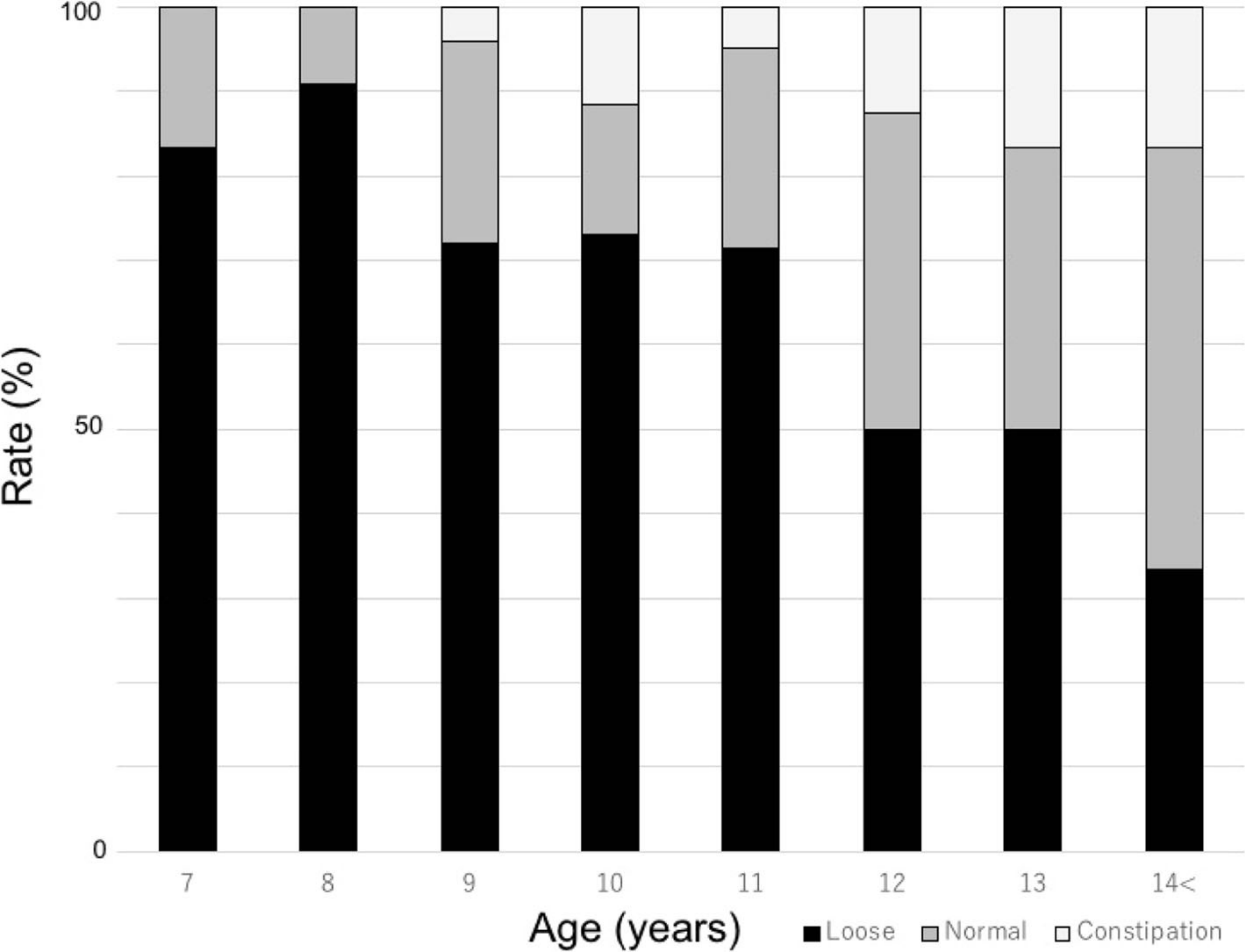
Stool form evaluation results in age group. Stool form was initially evaluated using the Bristol stool chart, and subsequently, each stool form category (loose, normal, and constipation) was designated on the criteria mentioned in the materials

The criteria of Bristol stool chart could confirm the 33.3–90.9% of loose stool rates in age groups (Fig. 1), and the overall loose stool rate was 69.4%. Breaking down the results of stool form evaluation into the scale levels of Bristol stool chart, which were as follows: constipation stool [scale 1 (4.2%) and 2 (3.5%)], normal stool [scale 3 (9.7%) and 4 (13.2%)], and loose stool [scale 5 (14.6%), 6 (31.3%), and 7 (23.6%)]. The loose stool rates declined as age increased, and the trend was statistically supported [adjusted odds ratio (AOR) 0.71, 95% confidence interval (CI) 0.59–0.86, *P* < 0.001] (Table 1).

**Table 1:** Risk factors associated with loose stool form in healthy Indonesian children

Factor	Prevalence (%)	Loose stool rate (%)	Adjusted odds ratio (95% confidence interval)	*P value
				(Wald’s test)
Age (years)	NA	NA	0.71 (0.59–0.86)	< 0.001*
Male (male vs. female)	NA	48/74 (64.9)	0.6 (0.28–1.28)	0.183
*Giardia intestinalis*	81/144 (56.3)	54/81(66.7)	0.78 (0.36–1.67)	0.520
*G. intestinalis* Assemblage A	23/144 (16.0)	16/23 (69.6)	0.73 (0.28–1.96)	0.537
*G. intestinalis* Assemblage B	78/144 (54.2)	51/78 (65.4)	0.79 (0.37–1.69)	0.551
Any *Entameoba* spp.	76/144 (52.8)	51/76 (67.1)	1.09 (0.51–2.34)	0.817
*E. histolytica*	0	NA	NA	NA
*E. dispar*	10/144 (6.9)	8/10 (80.0)	1.74 (0.34–8.9)	0.507
*E. moshkovskii*	0	NA	NA	NA
*E. hartmanni*	45/144 (31.3)	31/45 (68.9)	2.81 (1.1–3.7)	0.026*
*E. coli*	64/144 (44.4)	41/64 (64.1)	0.96 (0.45–2.06)	0.915

* values significant with respect to a *P* value of 0.05

*NA*, not applicable

### Prevalences of protozoan parasites

The molecular screening revealed the prevalences of *G. intestinalis* [56.3%, ass. A (16.0%) and ass. B (54.2%)], *E. histolytica* (0%), *E. dispar* (6.9%), *E. moshkovskii* (0%), *E. hartmanni* (31.3%), and *E. coli* (44.4%) (Tables 1 and 2). Although the molecular screening also targeted the pathogenic *E. histolytica* and the potentially pathogenic *E. moshkovskii*, those could not be detected in this study.

**Table 2:** Prevalences of intestinal protozoan parasites in age groups

Infection or colonization	Positive sample numbers (prevalence %) in age groups (years)							
	7	8	9	10	11	12	13	14 <
	(*n* = 12)	(*n* = 22)	(*n* = 25)	(*n* = 26)	(*n* = 21)	(*n* = 8)	(*n* = 24)	(*n* = 6)
*Giardia intestinalis*	8 (66.7)	11 (50.0)	11 (44.0)	15 (57.7)	12 (57.1)	4 (50.0)	19 (79.2)	1 (16.7)
*G. intestinalis* ass. A	3 (25.0)	3 (13.6)	1 (4.0)	2 (7.7)	5 (23.8)	1 (12.5)	8 (33.3)	0
*G. intestinalis* ass. B	8 (66.7)	10 (45.5)	11 (44.0)	15 (57.7)	10 (47.6)	4 (50.0)	19 (79.3)	1 (16.7)
Any *Entamoeba*	8 (66.7)	9 (40.9)	11 (44.0)	16 (61.5)	7 (33.3)	7 (87.5)	14 (58.3)	4 (66.7)
*E. histolytica*	0	0	0	0	0	0	0	0
*E. dispar*	0	1 (4.5)	2 (8.0)	3 (11.5)	3 (14.3)	1 (12.5)	0	0
*E. moshkovskii*	0	0	0	0	0	0	0	0
*E. hartmanni*	3 (25.0)	6 (27.3)	5 (20.0)	10 (38.5)	4 (19.0)	3 (37.5)	11 (45.8)	3 (50.0)
*E. coli*	7 (58.3)	9 (40.9)	8 (32.0)	13 (50.0)	6 (28.6)	6 (75.0)	12 (50.0)	3 (50.0)

The loose stool rate in each protozoan positive group was as follows: *G. intestinalis* (66.7%) [ass. A (69.6%) and ass. B (65.4%)], *E. dispar* (80.0%), *E. hartmanni* (68.9%), and *E. coli* (64.1%) (Table 1).

### Correlations between loose stool form and protozoan infection or colonization

Results of logistic regression analysis of protozoan infections or colonizations for diarrhea (loose stool form) as outcome (Table 1) revealed no significant results in examined protozoans including the pathogenic protozoan infection of *G. intestinalis* (AOR 0.78, 95% CI 0.36–1.67) with those genotypes [ass. A (AOR 0.73, 95% CI 0.28–1.96) and ass. B (AOR 0.79, 95% CI 0.37–1.69)], except in *E. hartmanni* colonizations (AOR 2.81, 95% CI 1.1–3.7, *P* = 0.026).

## Discussion

### Assessment of stool form by the Bristol stool chart

The stool form evaluation targeting healthy population could address the loose stool condition, which means mild to severe diarrheal conditions in those individuals (Fig. 1). Observed declined proportion of loose stool rates with age provides an evidence for our previous estimation in the field that elder children are more likely to provide solid stools, and also is consistent with the result of reference publication, which confirmed the reliability of this method [15].

### Asymptomatic *G. intestinalis* infection

The asymptomatic features, such as a persistence of duration (as long as 4 months) and a mean duration of cysts excretion (7.18 weeks), for *G. intestinalis* infections were described [16]. On the other hand, the asymptomatic infections in ordinary people have not yet been elucidated outside the clinical sites. By assessing the stool form of healthy school children, the correlation between *G. intestinalis* infection and loose stool, which indicates mild diarrheal status, was evaluated. The loose stool rates among *G. intestinalis* infected individuals were almost of the same level as those in commensal non-pathogenic *Entamoeba* spp.-colonized ones, and the correlation between the mild diarrheal stool condition and *G. intestinalis* infection could not be confirmed (Table 1). Taking the results together, *G. intestinalis* may regard commensal protozoan parasite at least in children over 6 years old under the parasite-endemic condition. As was indicated in previous observations [6], the early exposure to *G. intestinalis* infection seems to establish a certain resistance to the onset of the diarrheal symptom, though the infection itself could not be prevented.

### Possible pathogenicity, related to the *E. hartmanni* colonization

Possible pathogenicity related to the *E. hartmanni* colonization was observed (Table 1); however, there is almost no paper that suggests the pathogenicity of *E. hartmanni* colonization. To the best of our knowledge, the only two references have reported the *E. hartmanni* cases of AIDS-related diarrhea [17] and reactive arthritis in HLA-B27-positive individual [18]. Although the latter case suggested *E. hartmanni* as a causal agent of reactive arthritis, in both cases, metronidazole treatment was quite effective to *E. hartmanni*, and the complete relief of those symptoms was successfully achieved. It might be noteworthy that this significance, which indicates pathogenicity of *E. hartmanni*, was not observed when we use only severe diarrheal stool group (scale 6 and 7 of Bristol stool chart) for the analysis. This means that even *E. hartmanni* possess a certain pathogenicity, though the level of pathogenicity might be limited to mild. Meanwhile, the presence of recently proposed potential pathogens such as *E. moshkovskii* and *D. fragilis* together with the possible pathogenicity of *E. hartmanni* emphasize the need of further study on pathogenicity evaluation for commensal protozoan parasites that have been neglected so far.

### Limitation of this study

Due to the lack of evaluation of viral and bacterial pathogens for diarrhea, the results of this study might be considered to be preliminary, and further in-depth research is clearly required to understand the findings.

## Conclusions

We molecularly examined occult protozoan infections in healthy school children in Indonesia to evaluate the real pathogenicity of *G. intestinalis* and *Entamoeba* species. We found that only *E. hartmanni* colonization was associated with loose stool, though no correlation was found with *G. intestinalis* infection. In children living under a parasites-endemic area in Indonesia, *E. hartmanni* could be a mild pathogen, whereas *G. intestinalis* might be regarded as commensal, though the burden on children under 7 years of age may not be negligible.

## References

[1] Matey EJ, Tokoro M, Mizuno T, Matsumura T, Nagamoto T, Bi X, Oyombra JA, Sang WK, Songok EM, Ichimura H (2016). Positive correlation of HIV infection with *Giardia intestinalis* assemblage B but not with assemblage A in asymptomatic Kenyan children. AIDS.

[2] Quihui-Cota L, Astiazaran-Garcia H, Valencia ME, Morales-Figueroa GG, Lopez-Mata MA, Vazquez Ortiz F (2008). Impact of *Giardia intestinalis* on vitamin a status in schoolchildren from Northwest Mexico. Int J Vitam Nutr Res.

[3] Abou-Shady O, El Raziky MS, Zaki MM, Mohamed RK (2011). Impact of *Giardia lamblia* on growth, serum levels of zinc, copper, and iron in Egyptian children. Biol Trace Elem Res.

[4] Campos Ponce M, Incani RN, Pinelli E, Ten Kulve N, Ramak R, Polman K, Doak CM (2013). Are intestinal parasites fuelling the rise in dual burden households in Venezuela?. Trans R Soc Trop Med Hyg.

[5] Al-Shehri H, Stanton MC, LaCourse JE, Atuhaire A, Arinaitwe M, Wamboko A, Adriko M, Kabatereine NB, Stothard JR (2016). An extensive burden of giardiasis associated with intestinal schistosomiasis and anaemia in school children on the shoreline of Lake Albert, Uganda. Trans R Soc Trop Med Hyg.

[6] Goto R, Mascie-Taylor CG, Lunn PG (2009). Impact of intestinal permeability, inflammation status and parasitic infections on infant growth faltering in rural Bangladesh. Br J Nutr.

[7] Rogawski ET, Bartelt LA, Platts-Mills JA, Seidman JC, Samie A, Havt A, Babji S, Trigoso DR, Qureshi S, Shakoor S, Haque R, Mduma E, Bajracharya S, Gaffar SMA, Lima AAM, Kang G, Kosek MN, Ahmed T, Svensen E, Mason C, Bhutta ZA, Lang DR, Gottlieb M, Guerrant RL, Houpt ER, Bessong PO, Investigators M-EN (2017). Determinants and impact of *Giardia* infection in the first 2 years of life in the MAL-ED Birth Cohort. J Pediatric Infect Dis Soc.

[8] Shimokawa C, Kabir M, Taniuchi M, Mondal D, Kobayashi S, Ali IK, Sobuz SU, Senba M, Houpt E, Haque R, Petri WA, Hamano S (2012). *Entamoeba moshkovskii* is associated with diarrhea in infants and causes diarrhea and colitis in mice. J Infect Dis.

[9] Johnson EH, Windsor JJ, Clark CG (2004). Emerging from obscurity: biological, clinical, and diagnostic aspects of *Dientamoeba fragilis*. Clin Microbiol Rev.

[10] Matey EJ, Tokoro M, Nagamoto T, Mizuno T, Saina MC, Bi X, Oyombra JA, Okumu P, Langat BK, Sang WK, Songok EM, Ichimura H (2016). Lower prevalence of *Entamoeba* species in children with vertically transmitted HIV infection in Western Kenya. AIDS.

[11] O'Donnell LJ, Virjee J, Heaton KW (1990). Detection of pseudodiarrhoea by simple clinical assessment of intestinal transit rate. BMJ.

[12] Kamaruddin M, Tokoro M, Rahman MM, Arayama S, Hidayati AP, Syafruddin D, Asih PB, Yoshikawa H, Kawahara E (2014). Molecular characterization of various trichomonad species isolated from humans and related mammals in Indonesia. Korean J Parasitol.

[13] R. Core Team, R: a language and environment for statistical computing. R Foundation for Statistical Computing, Vienna, Austria, 2017. https://www.R-project.org/. Accessed 1 Dec 2018.

[14] Virasakdi Chongsuvivatwong, epiDisplay: Epidemiological Data Display Package, 2015. https://cran.r-project.org/web/packages/epiDisplay/index.html. Accessed 1 Dec 2018.

[15] Chumpitazi BP, Self MM, Czyzewski DI, Cejka S, Swank PR, Shulman RJ (2016). Bristol Stool Form Scale reliability and agreement decreases when determining Rome III stool form designations. Neurogastroenterol Motil.

[16] Mahmud MA, Chappell CL, Hossain MM, Huang DB, Habib M, DuPont HL (2001). Impact of breast-feeding on *Giardia lamblia* infections in Bilbeis, Egypt. Am J Trop Med Hyg.

[17] Rolston KV, Hoy J, Mansell PW (1986). Diarrhea caused by “nonpathogenic amoebae” in patients with AIDS. N Engl J Med.

[18] Schirmer M, Fischer M, Rossboth DW, Mur E, Dierich MP, Frischhut B (1998). *Entamoeba hartmanni*: a new causative agent in the pathogenesis of reactive arthritis?. Rheumatol Int.

